# Bacterial avidins are a widely distributed protein family in Actinobacteria, Proteobacteria and Bacteroidetes

**DOI:** 10.1186/s12862-021-01784-y

**Published:** 2021-04-09

**Authors:** Olli H. Laitinen, Tanja P. Kuusela, Sampo Kukkurainen, Anssi Nurminen, Aki Sinkkonen, Vesa P. Hytönen

**Affiliations:** 1 Faculty of Medicine and Health Technology, Tampere University, Tampere, Finland; 2 Horticulture Technologies, Natural Resources Institute Finland, Turku, Finland; 3 Fimlab Laboratories, Tampere, Finland

**Keywords:** Avidin, Phylogeny, Biotin-binding, Defense protein, Plant invasiveness

## Abstract

**Background:**

Avidins are biotin-binding proteins commonly found in the vertebrate eggs. In addition to streptavidin from *Streptomyces avidinii*, a growing number of avidins have been characterized from divergent bacterial species. However, a systematic research concerning their taxonomy and ecological role has never been done. We performed a search for avidin encoding genes among bacteria using available databases and classified potential avidins according to taxonomy and the ecological niches utilized by host bacteria.

**Results:**

Numerous avidin-encoding genes were found in the phyla Actinobacteria and Proteobacteria. The diversity of protein sequences was high and several new variants of genes encoding biotin-binding avidins were found. The living strategies of bacteria hosting avidin encoding genes fall mainly into two categories. Human and animal pathogens were overrepresented among the found bacteria carrying avidin genes. The other widespread category were bacteria that either fix nitrogen or live in root nodules/rhizospheres of plants hosting nitrogen-fixing bacteria.

**Conclusions:**

Bacterial avidins are a taxonomically and ecologically diverse group mainly found in Actinobacteria, Proteobacteria and Bacteroidetes, associated often with plant invasiveness. Avidin encoding genes in plasmids hint that avidins may be horizontally transferred. The current survey may be used as a basis in attempts to understand the ecological significance of biotin-binding capacity.

**Supplementary Information:**

The online version contains supplementary material available at 10.1186/s12862-021-01784-y.

## Background

The first known avidin was isolated from the chicken (*Gallus gallus*) egg white in 1941 [1] as a minor protein component showing extremely high avidity to biotin (K_d_ ≈ 10^−15^ M) and is a text-book example of tight protein–ligand interaction [1, 2]. This combined with the avidin’s compact tetrameric structure with four biotin-binding sites in each functional protein, and the existing methods to biotinylate a vast variety of biomolecules, has made avidin an important biotechnological tool in protein purification, detection, and assay technologies, but also in diagnostics and pharmaceuticals [3, 4].

The first bacterial avidin, *strept*avidin, was isolated from antibiotic-secreting *Streptomyces avidinii* bacteria in 1964 [5]. Since then, several new avidins have been experimentally verified from both eukaryotic and prokaryotic species. Ten avidin family members were identified in the chicken genome between the 1980s and the early 2000s [6, 7], and they were showed to resemble avidin structurally and functionally when expressed as recombinant proteins [8, 9]. Further eukaryotic avidins have been found in other avian species, reptiles, amphibians, sea urchin, fish, lancelet and fungi [10–12]. Several putative novel bacterial avidin genes have been detected from bacteria in a wide variety of environmental niches including symbiotic, marine, and pathogenic species. However, none of these bacterial avidins except streptavidin and closely related streptavidin v1 and v2 from *Streptomyces venezuelae* [13] have been confirmed to be expressed in nature. Avidins are made of beta barrels and their oligomeric state vary from loose dimeric assembly to very stable tetramer.

Avidin has been suggested to have antibiotic qualities, as it renders biotin vitamin unavailable. In oviparous animals, avidins are theorized to protect the eggs from microbes [14]. Evidence that chicken oviductal tissue produces avidin in response to bacterial, viral, and environmental stress supports this hypothesis [14–17]. A recent study revealed that avidin is expressed in avian primary gut epithelial cells along proinflammatory cytokines as acute phase proteins [18]. In line with these findings, two avidin genes, *Bjavd 1* and *2* [19] were found to be expressed in lancelet (*Branchiostoma japonicum*) in response to bacterial and heat shock stress. Interestingly, the Bjavd proteins appeared to recruit macrophages to the site of infection and thus acted as opsonins. While avidin has not been found in plants, transgenic avidin-expressing crops show resistance to insect pests [20, 21] and a correlation between biotin availability and root feeding nematodes was found in legume rhizosphere [22]. In fungi, the tamavidins (Tamavd 1 and Tamavd 2), discovered from the edible mushroom *Pleurotus cornucopiae*, have been suggested to protect from phytopathogenic fungi [23]. Simultaneously, biotin is essential cofactor avidin expression may cause negative effects. Known eukaryotic avidins are secreted proteins and this could be important factor to avoid the toxic effects. Reflecting the delicate balance in biotin availability, avidin-induced biotin deficiency causes low hatching success and teratogenicity in birds and mice, reflecting the toxic nature of avidin [24]. Silencing of zebavidin expression in zebrafish larvae using morpholinos did not reveal any significant changes in the early development of the fish [25]. Therefore, despite all the efforts, the exact biological role of avidins in various species is not fully understood.

Although avidin genes have been found in several bacterial clades, no comprehensive phylogeny of bacterial avidin sequences has been done. In this study, we present a phylogeny of the bacterial of avidins that were identified by screening Protein Data Bank, GenBank, The European Molecular Biology Laboratory (EMBL) Nucleotide Sequence Database, DNA Data Bank of Japan and UniProtKB databases using verified avidins as query sequences. We identified 946 protein and 213 nucleotide sequences corresponding to new putative avidins. In addition, we identified several new putative avidin clades, each showing their characteristic sequence features. Furthermore, we inspected the genomic and habitational context of the bacterial avidin family. Our results indicate that avidins are widespread among three bacterial phyla, and that the avidin-carrying bacteria inhabit several ecological niches and represent alternative lifestyles. This study reveals avidin family being very rich and proposes that avidin encoding genes are beneficial for bacteria in various environments.

## Results

### Avidins exist widely in bacteria

Queries were run against both protein and nucleotide databases with a set of nine verified avidin sequences. For the protein queries the amount of hits varied between 285 and 303, while for the nucleotide queries the amount of hits varied between 13 and 182. As the pooled query results contained a high amount of redundancy, the previously collected protein and nucleotide sequences were processed to obtain a cleaned-up set of unique 213 nucleotide and 946 protein sequences. This data together with the set of verified avidin sequences was used as a material for later analyses. Based on bacterial species information gathered via BLAST searches, we made a systematic analysis of bacterial genomes, and simplified the list of avidins by selecting representative avidins among groups of identical and highly similar proteins and associated them to representative bacterial species. This group was supplemented in the revision phase with 14 protein sequences, including representing putative avidins from Bacteroidetes. This resulted set of 118 different bacterial species are shown in Additional file 1: Table S1 and their sequences are listed in FASTA format in Additional file 2.

### Phylogeny, habitats, lifestyles and ecological significance of avidin harboring bacteria

**Fig. 1:**
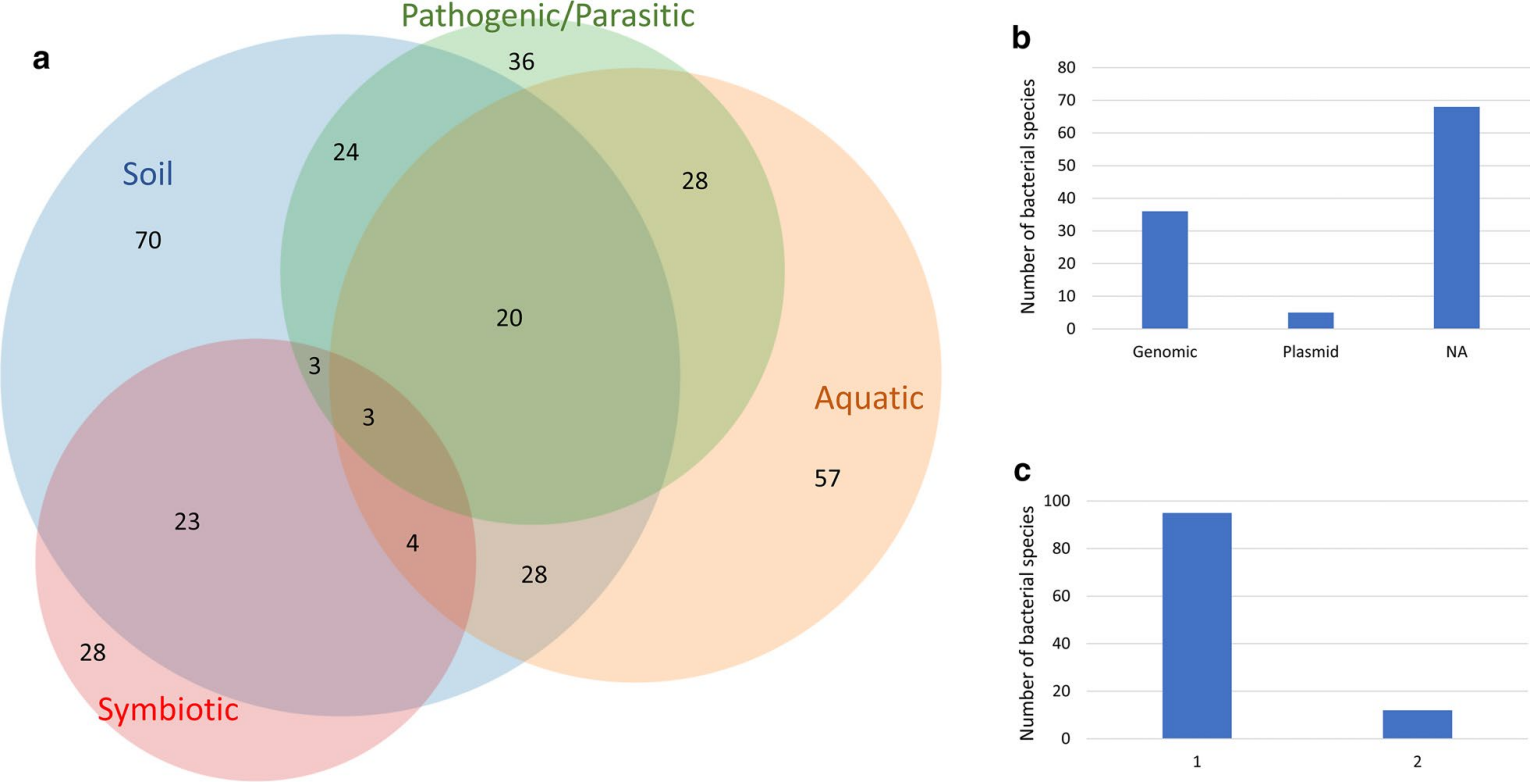
Overview on bacterial avidins. **a** Environmental niches of the bacterial species carrying putative avidins identified in this study. **b** Genomic location of the avidin genes. **c** Number of avidin gene copies. *NA* information not available

Those defined 118 bacterial species with putative avidins belong mainly in phyla Proteobacteria, Actinobacteria and Bacteroidetes with a single hit in phylum Synergistetes. In Actinobacteria, the most of the putative avidins belong to different *Streptomyces* species whereas in Proteobacteria the species are most often found within *Xanthomonas*, *Rhizobium*, *Bradyrhizobium*, *Burkholderia*, *Legionella*, *Methylobacterium* and *Mesorhizobium* (Additional file 1: Table S1). Despite coming mainly from two phyla, these new avidin-harboring bacteria show varied lifestyles and live in diverse environments. We approached the potential ecological significance of avidins by analyzing the lifestyles and environmental niches of those defined 118 avidin gene-carrying bacteria (Fig. 1a). Among this group, we observed many bacteria living in soil (70 species; 59% of species), while aquatic environments (57 species; 48%) were common habitats as well. Significant portion of these bacteria have interactions with either plants or animals. Previous studies have suggested that bacterial avidins may be involved in the competition between species as a part of the defense against other microbes or alternatively, as an agent controlling the root-feeding nematode composition [22]. In the present study avidin-carrying bacteria were often associated with mutualistic lifestyle with plants being either leaf endophytes or found from root nodule rhizosphere but also some plant pathogens causing bacterial canker and blight were identified (Additional file 1: Table S1). Bacterial avidin gene was observed in 36 species (31%), which are known or predicted human, fungus or plant pathogens. Human or animal pathogens were detected within avidin-carrying bacteria, potentially causing septicemia, pneumonia, melioidosis, pontiac fewer, glanders, cystic fibrosis, Crohn’s disease and lymphocytic leukemia (Additional file 1: Table S1). Interestingly, chemolithotrophic lifestyle was also found in *Cupriavidus* [26]. These results suggest that avidin expression provides advantage for bacteria with diverse lifestyles.

### Genomic association of avidin genes with other genes

We evaluated the genomic association between avidin genes and known biological pathways by inspecting the vicinity of avidin genes within bacterial genomes. This analysis revealed genes with multiple different functions being associated with avidin genes (Additional file 1: Table S2). Interestingly, avidin genes were residing in both plasmids (five identified cases) and in genomes (43 species) of the analyzed bacteria (Fig. 1b). Because > 10% avidin genes were detected within mobile elements, it is logical that genes responsible for DNA recombination were colocalized with avidin genes (Additional file 1: Table S2). This indicates that plasmid-encoded avidins can be transferred between different bacteria, and maybe even to other life forms too, via horizontal gene transfer. Thirteen bacterial species harbors more than one avidin gene (Fig. 1c), which further supports the importance of avidin for these bacteria. The enrichment analysis showed association with several DNA processing and mobile element GO-terms, which can correlate the plasmid origin of some of the identified avidins. Interestingly, two GO-terms statistically significantly associated with avidins included genes from defense pathways (Additional file 1: Table S2).

### Avidins falls into eleven phylogenetic clades

**Fig. 2:**
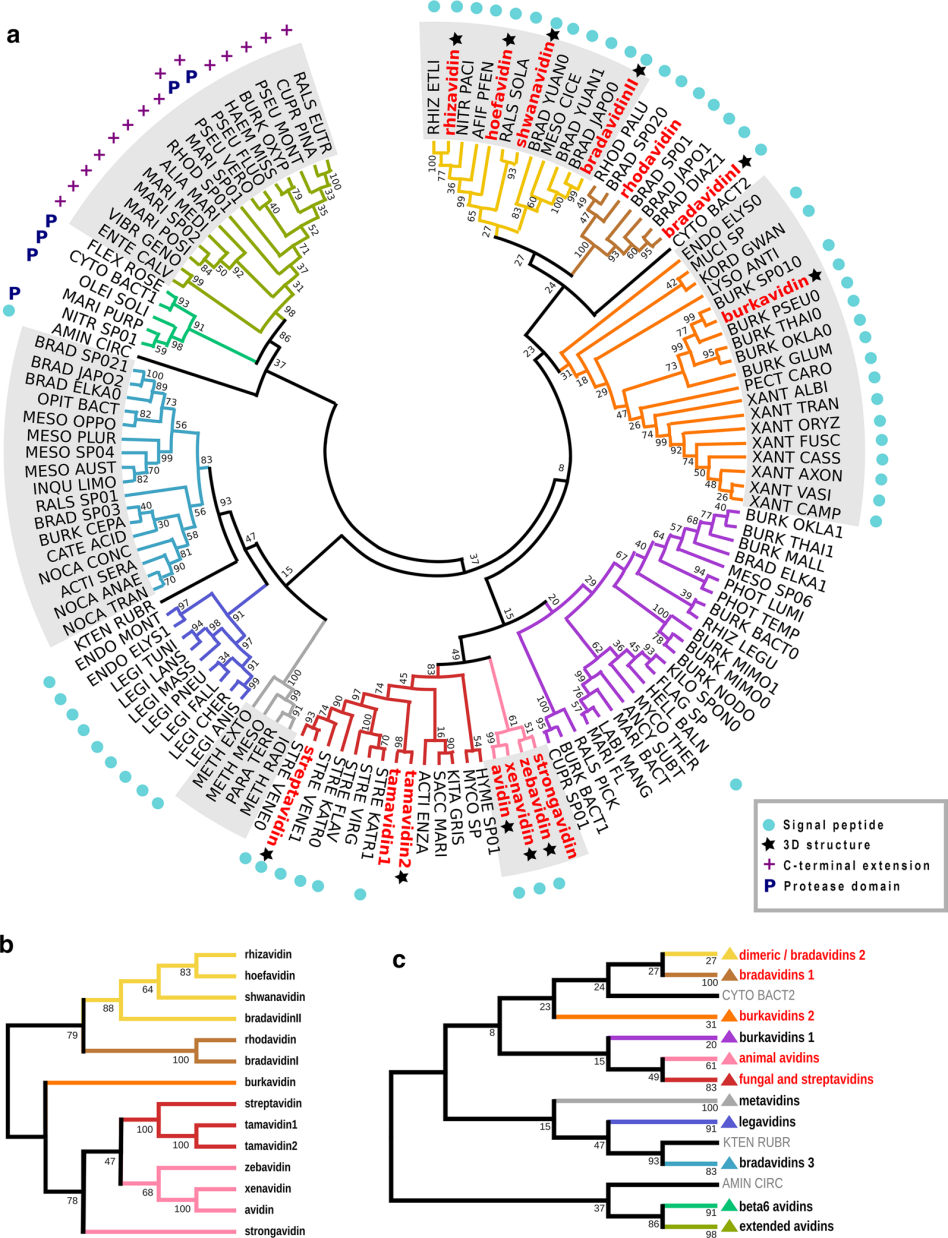
Phylogenetic analysis of putative bacterial avidins. **a** Phylogeny tree of the putative bacterial avidins. The verified avidins are shown with bold red font. The avidins with resolved 3D structure are indicated with black star symbol. The avidins containing predicted secretion signal peptide are indicated by cyan spheres. The avidins containing C-terminal extension are indicated by purple plus sign. The avidins containing predicted protease domain fusion are indicated by blue P letter. The bacterial avidins are grouped into 11 branches indicated with colors. **b** Phylogenetic cladogram tree of functionally verified avidins, colored according to **a**. **c** Phylogenetic cladogram tree of the verified and putative bacterial avidin sequences with collapsed subgroups. Triangle marks the collapsed clade, red text the clades containing verified avidins, and grey text that the indicated sequence was an outlier. The two outlier species, *Aminiphilus circumscriptus* and *Rhodonobacter* sp. OR444, were isolated from waste sludge and heavy metal polluted soil respectively

The phylogeny tree of the putative bacterial avidins (Fig. 2a) shows that the avidin family is highly divergent with 11 separate clades potentially representing structurally and functionally divergent avidin groups. For example, verified dimeric avidins (such as rhizavidin [27]) and avidins with ambivalent quaternary structure (such as bradavidin2, which appears to have a dynamic (transient) oligomeric state in solution depending on concentration [28]) clustered together into a clearly defined clade (Fig. 2a).

In order to evaluate the putative avidin sequence alignment and phylogeny tree, we also built a restricted phylogeny tree consisting only of the verified avidins (Fig. 2b). Several of the distinct clades within the comprehensive phylogeny (Fig. 2a) did not cluster together with clades containing verified avidin sequences, indicating that they potentially represent completely new avidin types (Fig. 2c). Avidins reported to have fungal origin, tamavidin 1 and tamavidin 2, clustered together with the rather well-defined clade of streptavidins. Meanwhile, the rest of the verified eukaryotic avidins formed a clade together. In this context, it should be noted that there would be a significant number of avidins in the genomes of eukaryotic species, not covered in this study.

Strongavidin was the only verified avidin that changed its position topologically, when the comprehensive phylogeny and the verified avidins’ phylogeny was compared. In the former, the strongavidin clustered together with avidins originating from animal species, meanwhile in the latter, it formed its own outgroup of the cluster including both streptavidins and eukaryotic avidins.

### Structure–function evaluation of the putative avidins

**Fig. 3:**
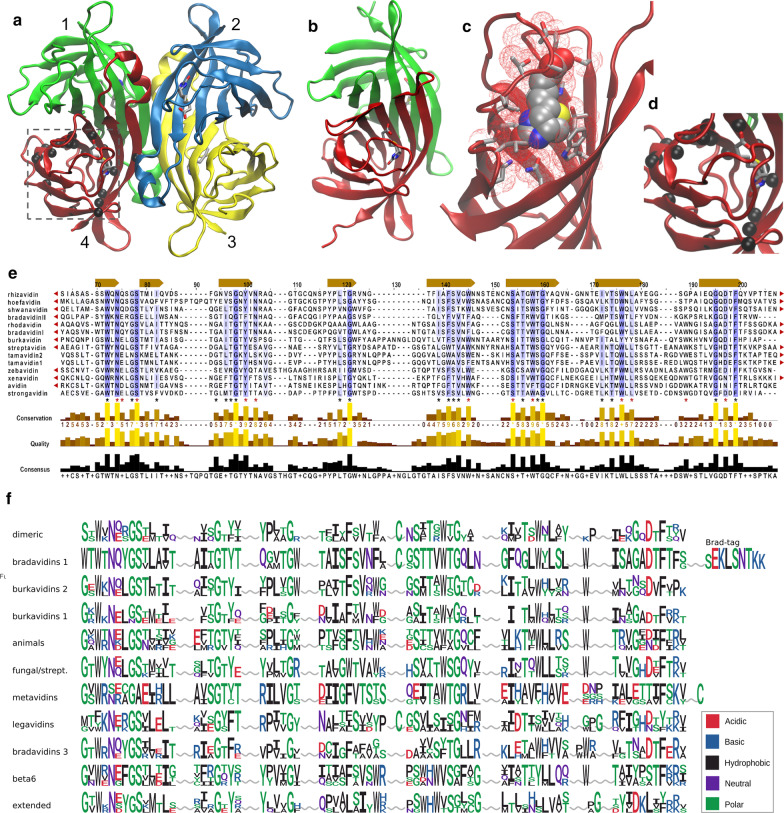
Characteristics of bacterial avidins. Avidins are made of beta barrels and their oligomeric state vary from loose dimeric assembly to very stable tetramer. **a** Structure of tetrameric chicken avidin with four bound biotin ligands (PDB 2AVI). The biotin molecules are represented as sticks and coloured according to the atom (C, gray; N, blue; O, red; S, yellow). The conserved residues indicated by black stars in **e** are indicated by black spheres, representing C-alpha atoms of residues 10, 15, 20, 27, 29, 30, 49, 51, 64, 66, 67, 68, 77, 80, 81, 93, 95, 116 and 120. **b** Structure of rhizavidin showing dimeric assembly (PDB 3EW2). **c** The biotin-binding site has very high structural complementarity with the ligand, represented here by chicken avidin monomeric subunit with bound biotin (PDB 2AVI). **d** Closer view of the area indicated in **a**. **e** Multiple sequence alignment of verified avidins. The black stars indicate highly conserved residues, which are also visualized in **a** and **d**. Red stars indicate residues in direct contact with the bound biotin ligand. Secondary structure elements (according to chicken avidin) are indicated by arrows above the alignment. **f** Groupwise sequence features of putative bacterial avidins. Sequence logos of the identified clades of the phylogeny tree of putative avidins were used to build sequence logos. Those logos were then aligned manually using the secondary structure elements as a guide. The residues are colored according to the chemical characteristics of the residues, as indicated in the legend

Avidin proteins are well-characterized structurally (Fig. 3a–d) and the functional role of the residues lining the ligand-binding site as well as residues within the subunit interfaces have been extensively studied in previously reported research, as reviewed by Laitinen et al. [3, 29]. Here, we present a structure-based multiple sequence alignment of the verified avidins (Fig. 3e), which could be used as a reference when inspecting the putative avidins. For example, there are a number of aromatic residues strongly conserved within putative avidins which have been found to be functionally important in previous studies [30–33]. Interestingly, only few positions remain completely conserved, when the whole landscape of the putative bacterial avidins is inspected using the sequence logo method (Fig. 3f). The first beta strand and the turn between the strands 1 and 2 shows higher conservation than the rest of the beta strands (Fig. 3f). The glycine residues within the strands 1, 2, 3, 4, and 6 are well conserved as are also the aromatic positions across the whole avidin sequence (Fig. 3f). These most likely reflect the strongly conserved beta-barrel structure of the avidin (Fig. 3a, d), having ligand-binding site lined up with aromatic residues in the middle of the barrel (Fig. 3c).

### Aspartic peptidase identified as a terminal fusion of Extended clade avidins

**Fig. 4:**
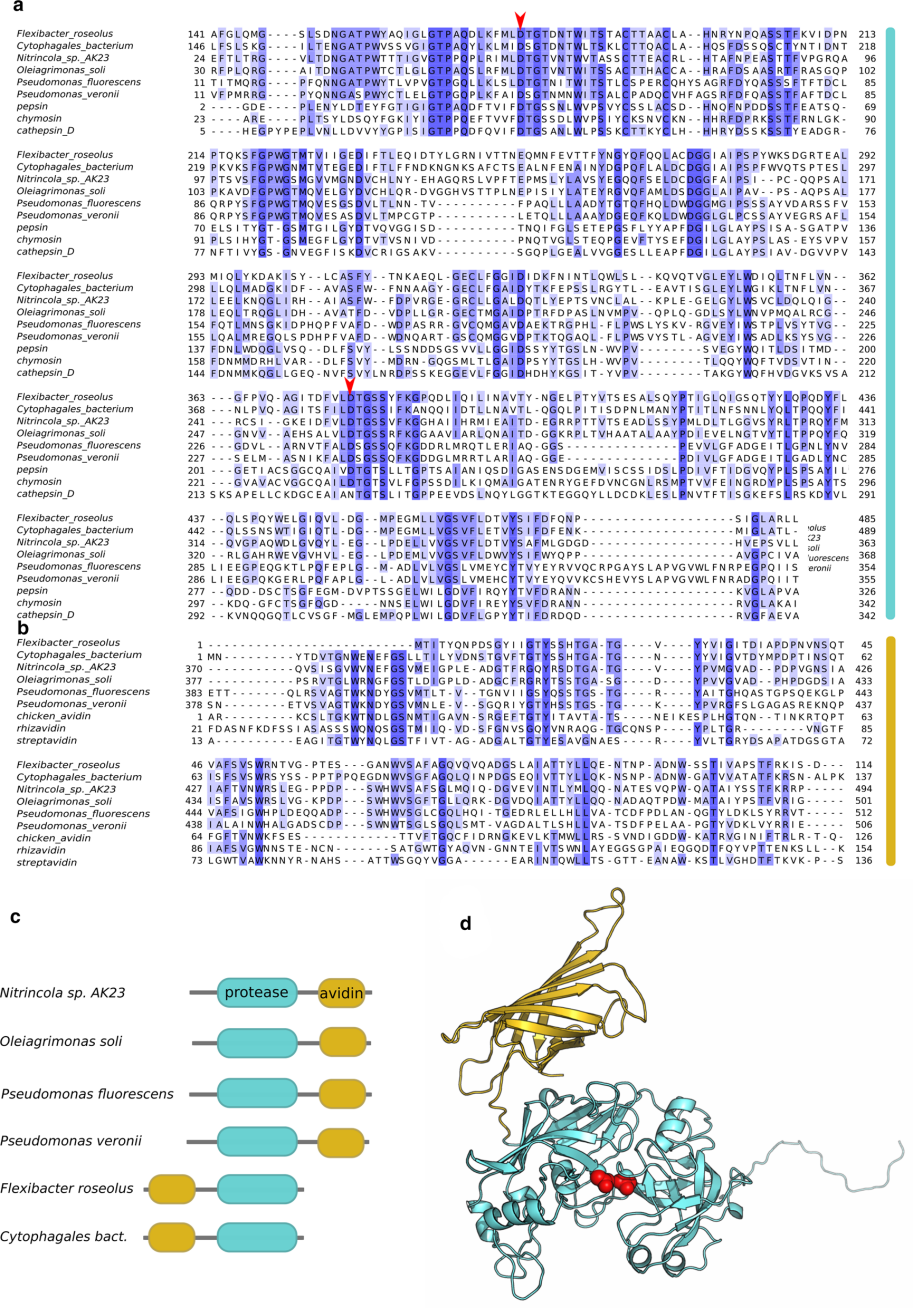
Bacterial avidins may be expressed as fusion proteins together with a pepsin-like aspartyl protease. **a** Multiple sequence alignment of the putative aspartyl protease domain of bacterial avidin sequences with the aspartyl proteases pepsin (*Sus scrofa*, PDB ID: 4PEP, [75]), cathepsin D (*Camelus dromedarius*, PDB ID: 4AA9, [82]) and chymosin (*Ixodes ricinus*, PDB ID: 5N71, [83]). The aspartic acid (asparagine in cathepsin D) residues of the putative active site are highlighted with red arrowheads [84]. **b** Multiple sequence alignment of the putative avidin domain of bacterial avidin sequences with streptavidin (*Streptomyces avidinii*, PDB ID: 3RY2, [76]), chicken avidin (Gallus gallus, PDB ID: 1VYO, [85]) and rhizavidin (*Rhizobium etli*, PDB ID: 3EW1, [53]). Multiple sequence alignment of the putative avidin domain of bacterial avidin sequences with streptavidin, chicken avidin and Xenopus avidin (xenavidin). Both alignments were carried out with T-Coffee in the Expresso mode (http://tcoffee.crg.cat/; [70, 80, 81]). **c** Schematic picture showing the domain organization of the putative protease-avidin fusion proteins. **d** Homology model of *Oleiagrimonas soli* protease-avidin fusion protein, generated with Modeller 9.25 [74]. Swine pepsin (PDB ID: 4PEP; [75]) was used as a template for the protease domain, and streptavidin (PDB ID: 3RY2; [76]) for the avidin domain. The active site aspartic acid residues are shown in red

Domain homology analysis with InterPro [34] detected a putative aspartic peptidase A1 family domain N-terminally of the putative avidin domain in two “Extended” clade pseudomonas sequences (*P. fluorescens* and *P. veronii*) and in *Oleiagrimonas soli, Cytophagales bacterium 1* and *Nitrincola nitratireducens* of the β6 clade (Fig. 4, Additional file 1: Table S3). In *Flexibacter roseolus* (β6 clade) an aspartic peptidase A1 family domain was predicted C-terminally of the putative avidin domain (Fig. 4). Aspartic peptidase A1 family, or pepsin-like aspartyl peptidases, are bilobed endopeptidases that have been previously found in bacteria [35]; we are however not aware of avidins having been previously reported to be connected to bacterial aspartic endopeptidases. Shorter (~ 150 residues) C-terminal extensions were found in several species in the “extended” subgroup: *Enterovibrio calviensis*, *Pseudomonas monteilii*, *Haematobacter missouriensis*, chemolithotrophs *Cupriavidus pinatuboensis* and *C. necator* (formerly *Ralstonia eutropha*), *Rhodanobacter* sp. (outlier grouped together with extended and β6), *Aliagarivorans marinus*, as well as *Marinomonas posidonica*, *M. mediterranea* and *Marinomonas* sp. MWYL1. The shorter extension appeared to be partial in *Burkholderia oxyphila* and *Maricaulis* sp. The shorter extensions were somewhat conserved (not shown), but InterPro and NCBI Conserved Domains Database search failed to identify conserved domains in the region.

### Plant-associated bacterial avidins

Based on our survey, several taxonomically distant Leguminous plant species host bacteria having genes encoding avidins. The species include significant agricultural plants species like common bean, soybean and peanut (Table 1). The other set consists of species with invasive characteristics outside their native areas. Sinkkonen et al. [22] have previously proposed that Leguminous plants benefit from the biotin-binding characteristics of their avidin-producing root symbionts. A probable reason is that these provide protection against root herbivory [22]. Our observation of the geographic distribution of crop and non-invasive wild plants with unintentionally sequenced bacterial avidins further supports this hypothesis.

**Table 1:** The economic significance and native distribution of plants known to host nitrogen-fixing root nodule bacteria with verified avidin production

Bacterial species	Plant species	Economic significance	Native distribution
*Bradyrhizobium arachidis*	Peanut	Crop plant	Southern America
*B. diazoefficiens*	Soybean	Crop plant	East Asia
*B. elkanii*	Green bean, soybean	Crop plant	South America, East Asia
*B. japonicum*	Soybean	Crop plant	East Asia
*B. pachyrhizi*	Mexican yam bean	Crop plant	Central America
*B.* sp. WSM2793	Rhynchosia totta	Native vine	Southern Africa
*B.* sp. WSM3983	Coral vine	Native vine	West and South-West Australia
*B.* sp. WSM4349	California broom	Native bush	California
*Burkholderia cepacia*	Yellow lupine	Crop plant	Eastern Mediterranean area

### Bacterial avidins in aquatic environments

With a single exception, bacteria carrying putative avidins found within Bacteroidetes belonged to species characterized in aquatic environments (Additional file 1: Table S1). *Ancylomarina* and *Labilibaculum* are genera present in anoxic coastal sediments and in anoxic waters of salt marshes and the Black Sea [36–40]. *Aquimarina* is a genus containing aquatic bacteria widely observed in salty waters [41]. *Flagellimonas* are freely moving bacteria found mainly in marine environments [42], and *Flexibacter roseolus* was isolated from a hot spring [43]. The sole known species of *Ekhidna* forms colonies on marine agar [44], and *Kordia periserrulae* was isolated in a digestive tract of a marine Eukaryote [45]. Today, genera *Fabibacter* and *Marinifilum* contain only marine organisms [46, 47]. Hypothetically, the ability to produce avidins might reduce browsing by predators of many of these easily harvestable organisms. Alternatively, in case of *Aquimarina*, *Ekhidna* and *Fabibacter*, avidin production might enhance pathogenesis; the genera are known to grow on aquatic Eukaryotes. Other taxa in Bacteroidetes were characterized at a taxonomically broad level. In addition to marine and aquatic species, hits within Bacteroidetes contained individual bacterial species from terrestrial ecosystems [48].

## Discussion

The first members of avidin protein family were isolated from very different life forms i. e. eukaryotic egg-laying bird, chicken, and soil living prokaryotic bacteria *Streptomyces avidinii* [1, 5]. Although the functional properties as well as quaternary and tertiary structures of these two proteins are well conserved [29], the low primary structure similarity (≈ 30%) raised a question if they have a common ancestor or if they have developed independently. While the catalogue of avidins has rapidly expanded, the observed sequence diversity has remained high. The same observation concerns the putative avidins characterized in this work. The overall sequence identity or similarity of the identified new avidins (Additional file 1: Table S4) reside in the twilight zone between major clades, which challenged the generation of high-quality alignment and phylogenetic tree. This suggests that if all identified avidins share a common ancestor, the avidin protein has a long evolutionary history.

Phylogenetic characterization of verified and putative avidins (Fig. 2) indicate that the known experimentally verified avidins are distributed along several different clades of the phylogeny tree. The previously characterized avidins belong to the clades of Dimeric avidins, Bradavidins1, Burkavidins2, Fungal and streptavidins and animal avidins. Additionally, completely new clades with a number of putative avidins were identified. Do those novel clades represent functional avidins? This question can be addressed by inspecting the conservation of well-known functional amino acid residues, which has been visualized using sequence logos of the phylogenetic clades in Fig. 3f. In a general level, the new avidins in these clades seem to be biotin binders, although some Burkavidins2 clade members contain several conservative and some non-conservative substitutions in positions with high conservation among verified avidins.

Fibropellins offer an interesting reference for the prediction of the biotin-binding activity of the putative avidins, as fibropellins do not bind biotin [49]. We have previously shown that by simultaneous mutation of only two biotin-binding residues of chicken avidin according to fibropellin template, i.e. substitution Trp110 with Lys and Trp70 with Arg, was enough to virtually demolish the avidin’s biotin-binding activity [31]. This indicates that one effective way to reduce biotin-binding capacity is a substitution of hydrophobic ligand-binding residues with bulky charged ones. Another way to lead to lower biotin binding is to replace residues forming hydrogen bonds with biotin by small hydrophobic residues or to introduce bulky residues to fill the biotin-binding pocket [29, 50].

Out of the new avidin groups, Burkavidins1 have considerably high number of non-conservative substitutions in their biotin-binding residues, but none of those hit the key aromatic residues and others also seem to be benign, supporting the possibility that they are true biotin binders. One of the β6 avidins members, i.e. Flex rose avidin (*Flexibacter roseolus,* Additional file 1: Table S1), lacks the whole β-sheet 1 and the following three hydrogen bond -forming biotin-binding residues residing in Loop 1 within the confirmed avidins. Other two β6 avidin members contain these residues and all three show considerably well conservation within the other biotin-binding residues. Therefore, it is possible that the polypeptide segment in the case of Flex rose avidin is missing due to sequencing error and all three members of this clade are true avidins with retained biotin-binding capacity. In contrast, Metavidins show the most numerous non-conservative changes in their respective biotin-binding residues, questioning their ability to bind biotin. Other new avidins in clades Legavidins, Bradavidins3 and extended avidins all look potent biotin binders. However, as learned from fibropellins, it is not easy to predict a degree of changes in biotin-binding residues to reliably judge, which of these new putative avidins really bind biotin without biochemical characterization. Also, interface residues, which define the strength and the presence of oligomeric assembly have effects on the ligand binding characteristics [31, 51, 52].

Overall, the sequence analysis of the putative and verified avidins reveals that there are only few highly conserved residues along the whole sequence, while some positions are semi-conserved. We used known structure of chicken avidin to inspect the location of the conserved residues, which are not directly linked to biotin binding (Fig. 3a, d). This analysis indicates that significant portion (> 10) of the conserved residues are located in the interface between subunits 1 and 4 while only one of them (Gly116 in the case of chicken avidin), is contributing to the interface between subunits 1 and 2. This suggests that the interactions supporting 1–4 dimer, analogous to those observed, for example, in rhizavidin [53], are more conserved within bacterial avidins than the interactions maintaining the tetrameric assembly observed in avidins from eukaryotic origin and in streptavidin.

Without experimental work, it is impossible to judge the functional nature of the novel avidin clades. As opposed to the high structural similarity of founding members of the avidin family, chicken avidin and streptavidin, the previous experimental work has revealed, that the avidin family is rather divergent in terms of structural details. For example, rhizavidin and hoefavidin [53, 54] utilize unique structural solution to build the tight biotin binding and this enables high biotin-binding affinity without contribution from the neighboring subunit, which appears absolutely necessary for the high biotin-binding affinity in the case of chicken avidin and streptavidin [31, 32]. The more thorough examination and discussion is found in the master’s thesis work by Tanja Kuusela (https://tampub.uta.fi/handle/10024/102386).

Avidins have not identified so far to contain other parts having functions on their own. Streptavidin has a C-terminal extension in its protein sequence, but it is cleaved in the mature form of the protein. Bradavidin has a C-terminal extension functioning as an intrinsic ligand [55] and biotin-binding protein B has a predicted C-terminal alpha-helix with no known function [6]. In this regard, the aspartic peptidase domain recognized in extended avidins is a novel finding that may be connected to avidin’s defence function.

Previous studies with birds suggest that oviparous vertebrates utilize avidins to fight against pathogenic organisms. For example, avidin expression has been induced with bacterial and virus infection in chicken [16, 17]. It is possible, that bacteria also utilize avidins to compete with other organisms and this has significance in bacterial pathogenesis. This is supported with the fact that streptavidin was originally identified as secreted antibiotic factor [5]. Our present study reveals that several common human pathogens carry genes encoding putative avidins. This raises a question whether biotin binding leads to more efficient invasion of host tissues due to reduced anti-inflammatory activity by eukaryotic, multicellular host organisms. Indeed, the life strategy of several human, fungal and plant pathogens seems to include potential for biotin binding (Fig. 1 and Additional file 1: Table S1). Another evolutionary reason for avidin production in pathogenic bacteria may be that biotin binding helps to outcompete other micro-organisms utilizing the same host or anatomic site, such as wound or enteral surface. Significant portion of the identified putative avidins (~ 50%, Fig. 2a) contain signal peptide for secretion, which would enable to avoid toxicity for the host cell. Finally, as several pathogens utilize also decaying tissues, avidins may protect from predation by microscopic multicellular organisms, such as nematodes [22].

Evaluation of plant association of bacterial avidins revealed several invasive plant species. Exotic leguminous invaders that host *Bradyrhizobium* spp. or *Burkholderia* spp. are a world-wide problem: Alien *Lupinus* spp. are serious exotic weeds in Europe, Australia and South America [56], http://www.NOBANIS.org), Australian *Acacias* are serious invaders in other parts of the world [57, 58], European Scotch broom (*Cytisus scoparius* L.) has formed large monocultures in Eastern Australia, New Zealand and North America [59], and South American *Mimosa pigra* L. has outcompeted natural vegetation in many ecosystems at other continents [60]. Main root symbionts of *M. pigra* are *Burkholderia* spp. [61], while *Bradyrhizobium* and other Rhizobiales prevail in the other invasive genera in novel geographic environments [56, 62]. In Australia, nitrogen fixing symbionts of *M. pigra* have a broader host range and a distant genetic relationship to strains isolated within the species’ indigenous region in South America [63]. Similarly, invasive *Fabaceous aliens* in New Zealand are nodulated by *Bradyrhizobium* species, while native legumes host a diverse nodulating bacterial fauna but not *Bradyrhizobium* sp. [64]. All these exotic leguminous species host bacteria that have been connected to the production of biotin-binding bacterial avidins. The findings lend support to the hypothesis by Sinkkonen et al. [22] that legumes may turn out to become invasive species outside their native region as they host bacteria producing biotin-binding compounds.

This study identified putative bacterial avidins as taxonomically and ecologically diverse group mainly found in Actinobacteria, Proteobacteria and Bacteroidetes. Because we had only limited number of experimentally verified avidins available, the obtained species coverage may evolve once more sequencing and proteomics data is available and when novel avidins have been functionally verified.

We identified that avidin genes are often localized in mobile genetic elements. Proposing avidins to function as defensive tools within bacteria closes the circle: Streptavidin was originally detected as antimicrobial agent secreted by *Streptomyces avidinii* [5]. We therefore postulate that avidins are widely distributed within bacteria and are functionally important tools for bacteria to defend their environmental niche, invade into other organisms, cause pathogenicity and help plants to invade. It is 80 years since the identification of chicken avidin but the story of avidins seems just to begin.

## Conclusions

Avidins are likely an old protein family and show high divergence across the bacteria. In general, avidins appear to be carried out by bacteria that inhabit niches in close intimacy of other bacteria, animals, fungi and/or plants. However, this could reflect bias from human interest, as these kinds of species are often research targets for their importance as beneficial, parasitic or pathogenic agents.

Apparently, there are only few strictly conserved features defining avidin, instead the different avidins seem to share approximately the same number of features from the pool of important sequence characteristics. The genomic context of avidin suggests importance for the bacteria, as the avidin gene was present on the primary chromosome more often than in secondary replicons. However, no clear association with genes of distinctive biological processes and pathways were present.

## Methods

### Database searches to identify novel bacterial avidin sequences and sequence processing

Nine verified avidin sequences: streptavidin (UniProtKB: P22629); bradavidin I (Q89IH6); bradavidin II (Q89U61); rhizavidin (Q8KKW2); shwanavidin (Q12QS6); avidin (P02701); zebavidin (E7F650); xenavidin (A7YYL1); and tamavidin 1 (B9A0T6), were used as the query sequences using the domain enhanced lookup time accelerated basic local alignment search tool (DELTA-BLAST) algorithm. Non-redundant protein databases were used as a search set including RefSeq, Protein Data Bank (PDB), GenBank, and UniProtKB [65]. The search was limited to bacteria and the maximum target sequence limit was set to 5000 with BLOSUM62 as the scoring matrix, and the parameters were set to adjust for short input sequence. The query was further refined with PSI-BLAST algorithm with E-value cut-off of 0.01 and required identity greater than 19% [66]. Nucleotide sequences were searched for with tBLASTn algorithm limited to bacteria against all non-redundant databases including Genbank, The European Molecular Biology Laboratory (EMBL) Nucleotide Sequence Database, and DNA Data Bank of Japan (DDBJ) [67–69] with the same search parameters as with protein queries. Duplicate sequences were removed with Python (3.4) language’s Biopython package and sequences corresponding to synthetic proteins or modified organisms were removed. All protein sequences were inspected to retrieve the original genomic features and their full nucleotide sequences. Similarly, the genomic position for each nucleotide sequence was obtained from genome tBLASTn and the partial DNA sequences were replaced with a previously annotated full cDNA feature, if such was present. The nucleotide sequences shorter than 300 bp were also extended from the genomic context if possible. Those nucleotide sequences that did not yet have a corresponding protein sequence were translated and added to the protein set. The list of 118 avidins used in the detailed analyses are provided in FASTA format in Additional file 2.

### Multiple sequence alignment

Two multiple sequence alignments (MSA) were constructed from the two different sequence sets. Structural MSA used the set of verified avidins, while a more comprehensive MSA was built upon the larger set of the putative avidins identified in this study. The structural MSA was constructed from the set of 14 verified avidins with T-Coffee in Expresso-mode [70]. The structures in the structural MSA construction were 1vyo for AVD, 4dne for Streptavd, 2y32 for Bradavd I and Rhodavd, 4ggz for Bradavd II, 3ew2 for Rhizavd, 3szj for Shwanavd, 4z6j for Hoefavd, 2uz2 for Xenavd, 4bj8 for Zebavd, 2fhl for Strongavd, 2szc for Tamavd 1 and Tamavd 2. MSA was cleaned up manually with AliView [71] by removing gaps from the unaligned N- and C-terminal termini. The alignment of the putative avidin sequences was constructed using the structural MSA of verified avidins as seed alignment with MUltiple Sequence Comparison by Log-Expectation (MUSCLE; [72]) to align the putative avidins against the profile of verified avidins. The set of putative avidin sequences was refined iteratively after aligning the full set by removing the short or highly similar sequences as well as highly variable sequences. This MSA was inspected with AliView and the gaps close to sequence termini were removed and the positions of biotin-binding and conserved AA homologues were used to adjust the MSA. The alignments were visualized using Jalview 2.

### Phylogenetic analyses

Phylogenetic analysis was performed in MEGA6.0 using the structural and full MSA, respectively [73]. The maximum likelihood (ML) algorithm was used with following parameters: Jones–Taylor–Thornton (JTT) model adjusted for site-specific AA sequences as the substitution model and the phylogeny quality was tested with bootstrapping (BTSP) with 300 replications, rates among sites were set gamma distributed with invariant sites, gaps or missing data was handled with partial deletion while site coverage cut-off was set to 95%, branch swap filter was strong, and ML heuristic method used the Nearest-Neighbour-Interchange (NNI) with initial tree calculated with the default neighbour-joining (NJ) method.

### Enrichment analysis

The following bacterial genomes, representing different sub-branches of the phylogenetic cladogram trees, were chosen to be assessed in enrichment analysis: *Bradyrhizobium diazoefficiens* (BA000040, GenBank), *Ralstonia eutropha* (CP000090–93), *Rhizobium etli* (CP001074–77), *Methylobacterium extorquens* (CP001298–1300), *Catenulispora acidiphila* (CP001700), *M. mediterranea* (CP002583), *Ralstonia pickettii* (CP00667–69), *Legionella pneumophila* (CR628336–38), and *Xanthomonas fuscans* (FO681494–97) [68]. The genomic features from these organisms and their assemblies were pooled together, and avidin (putative or verified) gene’s vicinity was defined as 500 bp upstream and downstream from the gene’s termini. Gene Ontology (GO-terms) were searched for each feature. If the feature was not annotated to any GO-term, the annotations for PFAM, IPR, or TGRFAM terms were mapped to corresponding GO-terms. Fischer’s exact test was performed to evaluate, if features annotated to a certain GO-term clustered significantly more often with avidin gene than expected by random distribution. Biopython was used for the processing and analysing the data.

### Visualization

The 3D structures obtained from Protein Data Bank were visualized using VMD 1.9.3.

### Homology modelling

The homology model of *Oleiagrimonas soli* protease-avidin fusion protein was generated with Modeller 9.25 [74]. Swine pepsin (PDB ID: 4PEP; [75]) was used as a template for the protease domain, and streptavidin (PDB ID: 3RY2; [76]) for the avidin domain.

### Pairwise similarity and identity

Pairwise sequence identity and pairwise sequence similarity were calculated using MatGAT 2.0 program (Matrix Global Alignment Tool) [77].

### Signal peptide prediction

The presence of signal peptide was predicted using SignalP 5.0 [78].

### Sequence logos

The sequence logos shown in Fig. 3f were built using ggseqlogo package in R [79]. The logos were manually curated to show only residues with occurrence above 20%.

## Supplementary Information

**Additional file 1: Table S1.** Representative bacterial avidins. **Table S2.** Most significantly enriched pathways among the genes in direct vicinity of avidin gene. **Table S3.** Prediction of the structure–function of extended avidins. **Table S4.** Pairwise identities for the representative avidin sequences.

**Additional file 2**. Bacterial avidin sequences in FASTA format.

## Data Availability

The datasets used and/or analysed during the current study are available from the corresponding author on reasonable request.

## Abbreviations

MSA: Multiple sequence alignment
ML: Maximum likelihood
JTT: Jones–Taylor–Thornton
NJ: Neighbour-joining
NNI: Nearest-Neighbour-Interchange
GO: Gene Ontology
