# Supplementary material for: Bacterial avidins are a widely distributed protein family in Actinobacteria, Proteobacteria and Bacteroidetes
Source: BMC Ecol Evol. 2021 Apr 9;21:53. doi: 10.1186/s12862-021-01784-y (PMC8033661; doi:10.1186/s12862-021-01784-y)
Supplement: Supplementary file 1 — Additional file 1: Table S1. Representative bacterial avidins. Table S2. Most significantly enriched pathways among the genes in direct vicinity of avidin gene. Table S3. Prediction of the structure–function of extended avidins. Table S4. Pairwise identities for the representative avidin sequences. [file 12862_2021_1784_MOESM1_ESM.docx]

**Supplementary materials** Laitinen et al.

**Bacterial Avidins Are a Widely Distributed Protein Family in Actinobacteria, Proteobacteria and Bacteroidetes**

Table S1 Representative bacterial avidins.

Table S2 Most significantly enriched pathways among the genes in direct vicinity of avidin gene.

Table S3 Prediction of the structure-function of extended avidins.

Table S4 Pairwise identities for the representative avidin sequences.

Supplementary references

**Table S1** Representative bacterial avidins.

| **Source organism** | **Environmental niche** | **Interspecies relations** | **Genomic location/ No. of recognized genes** | **Source** | **NCBI ID** |
| --- | --- | --- | --- | --- | --- |
| *Actinokineospora enzanensis* | soil | elephant ear skin flora, plant rhizosphere | -/1 | BioSample Accession: SAMN02441015 | WP_018681818.1 |
| *Actinocatenispora sera* | soil | human and animal microflora, endosporulation | -/1 | DOI: 10.1099/ijs.0.65270-0 | WP_030448363.1 |
| *Afifella pfennigii* | brackish water, benthic zone | microbial mat participant | -/1 | www.bacterio.net Search: afifella | WP_034491709.1 |
| *Aliagarivorans marinus* | marine | - | -/1 | DOI: 10.1099/ijs.0.008235-0 | WP_035480156.1 |
| *Aminiphilus circumscriptus* | waste sludge | - | -/1 | DOI: 10.1099/ijs.0.63614-0 | WP_029165400.1 |
| *Ancylomarina subtilis* | coastal sediment | - | genomic/1 | DOI: 10.1099/ijsem.0.001342 | WP_130306460.1 |
| *Bradyrhizobium diazoefficiens* | soil, nitrogen fixation | legume root nodular symbiont | genomic/2 | DOI: 10.1128/genomeA.01743-16 | WP_011084374.1, WP_011088404.1 |
| *Bradyrhizobium elkanii* | soil, nitrogen fixation | legume root nodular symbiont | -/2 | PMID: 8285723 | WP_038384200.1, WP_028344587.1 |
| *Bradyrhizobium diazoefficien*s  (former *B. japonicum*) | soil, nitrogen fixation | legume root nodular symbiont | genomic/1 | DOI: 10.1099/ijs.0.049130-0; 10.1093/dnares/9.6.225 | WP_011084374.1 |
| *Bradyrhizobium japonicum* | soil, nitrogen fixation | legume root nodular symbiont | genomic/2 | microbewiki.kenyon.edu; Search: Bradyrhizobium japonicum | WP_018645840.1, WP_018646127.1 |
| *Bradyrhizobium sp. WSM1253* | soil, nitrogen fixation | legume root nodular symbiont | -/1 | DOI: 10.1186/s40793-015-0115-9 | WP_007602433.1 |
| *Bradyrhizobium sp. WSM3983* | soil, nitrogen fixation | legume root nodular symbiont | -/2 | BioSample Accession: SAMN02440715 | WP_027528985.1, WP_156947219.1 |
| *Bradyrhizobium sp. STM* | soil, nitrogen fixation | legume root nodular symbiont | -/1 | BioProject Accession: PRJNA162993 | WP_008969504.1 |
| *Bradyrhizobium yuanmingense* | soil, nitrogen fixation | legume root nodular symbiont | -/2 | DOI: 10.1111/j.15746968.2008.01169.x | WP_036032120.1, WP_036015887.1 |
| *Bradyrhizobium diazoefficien*s  (former *B. japonicum*)  (**bradavidin, bradavidin2**) | soil, nitrogen fixation | legume root nodular symbiont | -/2 | DOI: 10.1016/j.bbapap.2008.04.010;  10.1074/jbc.M414336200 | WP_011088404.1,  WP_011084374.1 |
| *Burkholderiaceae bacterium* | soil, aquatic, voluntary dormancy | human and animal pathogen | genomic/2 | BioSample Accession: SAMN03340296 | KJK14170.1, KJK22176.1 |
| *Burkholderia cenocepacia* | soil, aquatic, aerosol | biofilm, opportunistic human pathogen, antibiotic resistant | genomic/1 | DOI: 10.1097/01.mcp.0000181475.85187.ed | CAR57556.1 |
| *Burkholderia glumae* | soil, aquatic, voluntary dormancy | plant pathogen | genomic/1 | DOI: 10.1094/PDIS-10-13-1024PDN | WP_012732748.1 |
| *Burkholderia mallei* | Animals, humans | human and animal pathogen | genomic/1 | DOI: 10.2174/157489107782497335 | AFR14207.1 |
| *Burkholderia mimosarum* | soil, nitrogen fixation | mimosa root nodular symbiont | genomic/2 | DOI: 10.1099/ijs.0.64325-0 | WP_028215459.1, WP_155837025.1 |
| *Burkholderia nodosa (Parabulkholderia nodosa)* | soil, nitrogen fixation | mimosa root nodular symbiont | genomic/1 | DOI: 10.1099/ijs.0.64873-0 | WP_028208921.1 |
| *Burkholderia oklahomensis* | soil, aquatic, voluntary dormancy | human and animal pathogen | genomic/2 | DOI: 10.1099/ijs.0.63991-0 | WP_010111286.1, WP_038800244.1 |
| *Burkholderia oxyphila* | soil, acidic | - | genomic/1 | DOI: 10.1099/ijs.0.017368-0 | WP_028223019.1 |
| *Burkholderia pesudomallei*  (**burkavidin**) | soil, aquatic, voluntary dormancy | human and animal pathogen | genomic/2 | DOI: 10.2174/157489107782497335 | ABA48099.1, AFR14207.1 |
| *Burkholderia thailandensis* | soil, aquatic, voluntary dormancy | human and animal pathogen | genomic/2 | DOI: 10.1128/JCM.01585-06 | WP_009895923.1, AVR09418.1 |
| *Catenulispora acidiphila* | soil, marine, fresh water | decomposition of organic materials | -/1 | DOI: 10.1099/ijs.0.63858-0 | WP_015795036.1 |
| *Cupriavidus pinatubonensis* | volcanic sludge, oxidation, metal resistance | - | genomic/1 | DOI: 10.1099/ijs.0.63922-0 | WP_140951620.1 |
| *Cupriavidus sp.*  *SK-3* | aquatic sediment, contaminant degradation | - | -/1 | DOI: 10.1128/genomeA.00664-14 | WP_035871165.1 |
| *Cytophagales bacterium* | aquatic | toxigenic freshwater cyanobacterial mats | -/? | DOI: 10.1038/s41396-019-0374-3; DOI: 10.1038/nbt.4229 | TAG59864.1, HAA17199.1 |
| *Endozoicomonas elysicola* | marine | sea slug endogastric flora, coral and sponge microflora | -/2 | DOI: 10.1016/j.syapm.2006.07.003;  10.3354/dao02636 | WP_020581719.1, KEI71898.1 |
| *Endozoicomonas montiporae* | marine | coral microflora | -/1 | DOI: 10.1099/ijs.0.014357-0;  10.3389/fmicb.2016.00251 | WP_034878095.1 |
| *Vibrio calviensis*  (former *Enterovibrio calviensis*) | marine | - | -/1 | DOI: 10.1099/ijs.0.001990-0;  10.1099/00207713-52-2-549 | WP_017016576.1 |
| *Flagellimonas sp. XY359* | aquatic | - | genomic/1 | img.jgi.doe.gov/cgi-bin/m/main.cgi?section=TaxonDetail&page=taxonDetail&taxon_oid=2873577346 | WP_136465220.1 |
| *Flexibacter roseolus (Hugenholzia roseola)* | aquatic | fish pathogen | -/1 | www.bacterio.net; Genus: Flexibacter | WP_086047884.1 |
| *Haematobacter missouriensis* | soil | possible sepsis causing human pathogen | -/1 | DOI: 10.1128/JCM.01188-06 | WP_143412891.1 |
| *Hellea balneolensis* | marine surface | - | -/1 | DOI: 10.1099/ijs.0.65424-0 | WP_084169061.1 |
| *Hoeflea phototrophica*  *(****hoefavidin****)* | marine, photosynthetic | - | -/1 | DOI: 10.1016/j.jsb.2015.06.020; 10.1099/ijs.0.63958-0;  10.4056/sigs.3486982; | WP_007199185.1 |
| *Hymenobacter sp. AT01-02* | desert soil, UV-resistance, Mn and Fe accumulation | - | -/1 | DOI: 10.1128/genomeA.01701-15 | WP_055562988.1 |
| *Inquilinus limosus* | aquatic | opportunistic human pathogen | -/1 | DOI: 10.3201/eid1103.041078  10.3201/eid1406.071355 | WP_034841399.1 |
| *Kiloniella spongiae* | marine | sponge microflora | -/1 | DOI: 10.1099/ijs.0.069773-0 | WP_047765324.1 |
| *Streptomyces griseolisporeus*  (former *Kitasatospora griseola*) | soil | secretes exotoxins, antibiotics and antifungal agents | -/1 | DOI: 10.1128/genomeA.00208-15 | WP_043911721.1 |
| *Kordiimonas gwangyangensis* | marine sediment | - | -/1 | DOI: 10.1099/ijs.0.63684-0 | WP_011088404.1 |
| *Labilibaculum manganireducens* | marine sediment | psychrotolerant, neutrophilic and halotolerant, reduces metals | -/1 | DOI: 10.3389/fmicb.2017.02614 | WP_101311097.1 |
| *Legionella anisa* | aquatic, requires Cys | plant pathogen, amoebae intracellular parasite | -/1 | DOI: 10.1371/journal.pone.0159726; 10.1128/aem.49.2.305-309.1985 | WP_019235159.1 |
| *Legionella cherrii* | aquatic, nitrogen fixation, requires Cys | human pathogen | -/1 | DOI: 10.1128/AEM.69.1.533541.2003 | WP_035901363.1 |
| *Legionella fallonii* | aquatic, aerosol, requires Cys | human pathogen | -/1 | DOI: 10.1099/00207713-51-3-1151 | WP_045095505.1 |
| *Legionella lansingensis* | aquatic | human pathogen | genomic/1 | PMID: 1401005 | WP_028372330.1 |
| *Legionella massiliensis* | aquatic | human pathogen | -/1 | DOI: 10.1099/ijs.0.037853-0;  10.1128/genomeA.01068-14 | CDZ76781.1 |
| *Legionella pneumophila* | aquatic, requires Cys | amoebae intracellular parasite | -/1 | DOI: 10.1078/1438-4221-0013 | ANN96495.1 |
| *Legionella tunisiensis* | aquatic | amoebae intracellular parasite | -/1 | DOI: 10.1099/ijs.0.037853-0 | WP_019215553.1 |
| *Lysobacter antibioticus* | soil, aquatic, extremophile | plant microflora, salamander skin microflora, secretes antibiotics and antifungal agents, probable legume root nodular symbiont | -/1 | DOI: 10.1007/s00284-009-9481-0;  10.3389/fmicb.2015.01243 | WP_161786125.1 |
| *Marinifilaceae bacterium T32 S1C* | marine sediment | - | -/1 | www.uniprot.org/proteomes/UP000282985 | RUT78256.1 |
| *Marinifilum sp. N1E240* | marine | - | -/1 | - | WP_152734636.1 |
| *Marinomonas mediterranea* | marine, contaminant degradation | probable obligate symbiont to other bacteria | -/1 | DOI: 10.1099/mic.0.26524-0;  10.4056/sigs.2545743 | WP_013662907.1 |
| *Marinomonas posidonica* | marine | seaweed microflora | genomic/1 | DOI: 10.4056/sigs.2976373 | WP_013796661.1 |
| *Marichromatium purpuratum* | marine, photosynthetic | - | genomic/1 | www.bacterio.net; Genus: Marichromatium | WP_005223961.1 |
| *Maricaulis sp. JL2009 (Euryhalocaulis caribicus)* | - | - | -/1 | DOI: 10.1128/genomeA.00407-13; Taxon ID (NCBI): 1161401 | WP_051122713.1 |
| *Marinomonas sp. MWYL1* | marine | salt marsh grass microflora | -/1 | genome.jgi.doe.gov; Search: Marinomonas MWYL1 | WP_012069352.1 |
| *Mesorhizobium australicum* | soil, nitrogen fixation | legume root nodular symbiont | genomic/1 | DOI: 10.1099/ijs.0.005728-0 | AGB46899.1 |
| *Mesorizobium plurifarum*  (former *M. ciceri*) | soil, nitrogen fixation | legume root nodular symbiont | genomic/1 | DOI: 10.4056/sigs.4458283;  10.1111/j.15746941.2009.00776.x | WP_197031631.1 |
| *Mesorizobium plurifarum*  (former *M. ciceri*) | soil, nitrogen fixation | legume root nodular symbiont | -/1 | DOI: 10.1371/journal.pone.0117667 | CDX43292.1 |
| *Mesorhizobium opportunistum* | soil, nitrogen fixation | legume root nodular symbiont | -/1 | DOI: 10.4056/sigs.4538264;  10.1099/ijs.0.005728-0 | WP_013894719.1 |
| *Mesorhizobium sp. LSJC280B00* | soil, nitrogen fixation | legume root nodular symbiont | -/1 | BioSample Accession: SAMN02359695 | WP_023677073.1 |
| *Mesorhizobium sp. LSJC255A00* | soil | - | -/1 | BioSample Accession: SAMN02359689 | EQC00282.1 |
| *Methylobacterium extorquens* | soil, aquatic sediment | opportunistic human pathogen | -/1 | DOI: 10.1371/journal.pone.0013001 | ACK86247.1 |
| *Methylobacterium mesophilicum* | soil | opportunistic human pathogen | plasmid/1 | DOI: 10.1086/313815; PMID: 8469180 | WP_083920243.1 |
| *Methylobacterium radiotolerans* | soil, radiation resistance | opportunistic human pathogen | -/1 | microbewiki.kenyon.edu  Genus: Metylobacterium; DOI: 10.1128/JCM.01241-11 | ACB28100.1 |
| *Mucilaginibacter sp. OV119* | soil | populus root rhizosphere | -/1 | www.uniprot.org/proteomes/UP000248452 | WP_110582673.1 |
| *Mycobacterium sp. BK086* | - | plant cell wall | -/1 | https://gold.jgi.doe.gov/analysis_projects?id=Ga0307707 | WP_133697041.1 |
| *Mycobacterium thermoresistibile* | soil, aquatic | human pathogen, found in mucous membranes, urine, gastric fluid | plasmid/1 | DOI: 10.1002/pro.2084; 10.1128/jcm.14.5.593-595.1981 | WP_003924221.1 |
| *Nitratireductor pacificus* | marine, denitrification | - | -/1 | DOI: 10.1099/ijs.0.024356-0 | EKF18360.1 |
| *Nitrincola sp. AK23 (Nitrincola nitrareducens)* | aquatic, alkaline | - | -/1 | DOI: 10.1016/j.syapm.2015.09.002 | WP_036510030.1 |
| *Nocardia anaemiae* | - | isolated from immunocompromized patient | -/1 | DOI: 10.3314/jjmm.46.21 | WP_062988101.1 |
| *Nocardia concava* | - | human pathogen | -/1 | DOI: 10.1099/ijs.0.63280-0 | WP_040805712.1 |
| *Nocardia transvalensis* | - | human pathogen | -/1 | DOI: 10.4103/0970-2113.99121 | WP_040748313.1 |
| *Oleiagrimonas soli* | soil, contaminant degradation | - | -/1 | DOI: 10.1099/ijs.0.000158 | WP_043104392.1 |
| *Parafilimonas terrae* | soil | - | -/1 | DOI: 10.1099/ijs.0.061945-0 | WP_076729952.1 |
| *Pectobacterium carotovorum* | soil, aquatic, aerosol, nitrogen fixation | insect endogastric flora, secretes antibiotics, plant pathogen | -/1 | DOI: 10.5423/PPJ.OA.12.2013.0117 | WP_071820981.1 |
| *Photorhabdus luminescens* | - | endosymbiont of entomopathogenic nematode, insect pathogen, used as toxin against the host insect by the nematode | genomic/1 | DOI: 10.1128/AEM.69.4.1890-1897.2003;  10.1099/00207713-49-4-1645; microbewiki.kenyon.edu | WP_052105826.1 |
| *Photorhabdus temperate* | - | endosymbiont of entomopathogenic nematode, insect pathogen, used as toxin against the host insect by the nematode | -/1 | DOI: 10.1128/genomeA.01273-14;  10.1099/ijs.0.2008/000273-0;  10.1099/00207713-49-4-1645 | EQC00282.1 |
| *Pseudomonas fluorescens* | soil, aquatic, nitrogen reducing | fungal pathogen, endosymbiont or endoparasite of amoebae, antifungal plant root symbiont, secretes antibiotics | -/1 | DOI: 0.1128/CMR.00044-14; microbewiki.kenyon.edu  Search: Pseudomonas fluroscens | WP_177077996.1 |
| *Pseudomonas monteilii*  (a.k.a. *P. fluorescens*) | soil, aquatic, nitrogen reducing | fungal pathogen, endosymbiont or endoparasite of amoebae, antifungal plant root symbiont, secretes antibiotics | -/1 | DOI: 10.1099/00207713-47-3-846 | WP_028699818.1 |
| *Pseudomonas veronii*  (a.k.a. *P. fluorescens*) | soil, aquatic, nitrogen reducing | fungal pathogen, endosymbiont or endoparasite of amoebae, antifungal plant root symbiont, secretes antibiotics | -/1 | DOI: 10.1128/genomeA.00258-13;  10.1186/s40793-016-0198-y | WP_017846356.1 |
| *Ralstonia solanacearum* | soil, voluntary dormancy | plant pathogen | -/1 | DOI: 10.1111/mpp.12038 | WP_080894517.1 |
| *Ralstonia eutropha*  (a.k.a. *Cupriavidus necator*) | soil, aquatic, contaminant degradation | - | genomic/1 | microbewiki.kenyon.edu,  Search: Ralstonia eutropha | WP_011301584.1 |
| *Ralstonia pickettii* | wet soil, aquatic sediment | biofilm, opportunistic human pathogen | genomic/1 | DOI: 10.1016/j.jhin.2005.08.015; microbewiki.kenyon.edu;  Search: Ralstonia pickettii | AGW89019.1 |
| *Ralstonia sp. UNC404CL21Col* | soil | plant hosted, forms communities | genomic/1 | BioProject Accession: PRJNA213749;  BioSample Accession: SAMN02743945 | WP_027681179.1 |
| *Rhizobium leguminosarum* | soil, nitrogen fixation | legume root nodular symbiont | plasmid/1 | DOI:  10.1111/j.1365294X.2004.02259.x | WP_029871419.1 |
| *Rhizobium etli* | soil, nitrogen fixation | legume root nodular symbiont | -/1 | DOI: 10.1128/AEM.69.2.884893.2003 | WP_012489429.1 |
| *Rhizobium etli*  (**rhizavidin**) | soil, nitrogen fixation | legume root nodular symbiont | plasmid/1 | DOI: 10.1042/BJ20070076 | WP_004674376.1 |
| *Rhodopseudo-monas palustris* | wet soil, aquatic sediment, nitrogen fixation, carbon fixation, photosynthetic | - | plasmid/1 | microbewiki.kenyon.edu,  Search: Rhodopseudomonas palustris | KIZ38333.1 |
| *Rhodopseudo-monas palustris*  (**rhodavidin**) | wet soil, aquatic sediment, nitrogen fixation, carbon fixation, photosynthetic | - | -/1 | DOI: 10.1371/journal.pone.0176086 | WP_011472104.1 |
| *Rhodanobacter sp. OR444* | soil, heavy metal resistance and purification | - | genomic/1 | DOI: 10.1128/mBio.02234-15 | WP_027492140.1 |
| *Saccharomono-spora marina* | marine sediment | - | -/1 | DOI: 10.1099/ijs.0.017038-0;  10.4056/sigs.2655905 | WP_009154511.1 |
| *Shewanella denitrificans (***schwanavidin***)* | marine, denitrification | species in same genus participate sponge microflora | genomic/1 | DOI: 10.1074/jbc.M112.357186 | WP_011495364.1 |
| *Streptomyces flavotricini* | soil, sporulation | secretes antibiotics and antifungal agents | genomic/1 | DOI: 10.1038/ja.2011.12; microbewiki.kenyon.edu,  Genus: Streptomyces | WP_075969886.1 |
| *Streptomyces katrae* | soil, sporulation | secretes antibiotics | -/1 | microbewiki.kenyon.edu, Genus: Streptomyces | WP_045950539.1 |
| *Streptomyces venezuelae* | soil, sporulation | secretes antibiotics | -/? | DOI: 10.1016/0167-4781(95)00077-t; DOI: 10.1128/genomeA.00337-16 | Q53532.1, Q53533.1 |
| *Streptomyces virginiae* | soil, sporulation | secretes antibiotics and antifungal agents | genomic/1 | DOI: 10.1099/00221287-136-3-581; microbewiki.kenyon.edu, Genus: Streptomyces | KOU30293.1 |
| *Streptomyces violaceus;* (**streptavidin**)  (former *S. avidinii*) | soil, sporulation | secretes antibiotics | -/1 | microbewiki.kenyon.edu, Genus: Streptomyces | WP_189973520.1 |
| *Vibrio genomosp.* | marine, bacterioplankton | secretes antibiotics | -/1 | BioProject Accession: PRJNA164825 | WP_017035909.1 |
| *Xanthomonas albilineans* | soil, aquatic | plant pathogen, opportunistic animal pathogen | -/1 | PMID: 20572987 | CBA15444.1 |
| *Xanthomonas axonopodis*  (former *X. cassavae*) | soil, aquatic | plant pathogen, opportunistic animal pathogen | genomic/1 | DOI: 10.1128/AEM.05189-11;  micorebwiki.kenyon.edu, Search: Xanthomonas axonopodis | WP_029218441.1 |
| *Xanthomonas campestris* | soil, aquatic | plant pathogen, opportunistic animal pathogen | genomic/1 | DOI: 10.1094/PHYTO.2001.91.5.4 92;  micorebwiki.kenyon.edu, Search: Xanthomonas campestris | WP_029220486.1 |
| *Xanthomonas axonopodis*  (former *X. cassavae*) | soil, aquatic | plant pathogen, opportunistic animal pathogen | genomic/1 | DOI: 10.1128/AEM.05189-11; micorebwiki.kenyon.edu,  Search: Xanthomonas axonopodis | WP_029218441.1 |
| *Xanthomonas Fuscans* | soil, aquatic | plant pathogen, opportunistic animal pathogen | genomic/1 | DOI: 10.1128/AEM.05189-11;  10.1186/1471-2164-14-761 | WP_022557741.1 |
| *Xanthomonas oryzae* | soil, aquatic | plant pathogen, opportunistic animal pathogen | genomic/1 | DOI: 10.1111/j.13643703.2006.00344.x | WP_014501819.1 |
| *Xanthomonas translucens* | soil, aquatic | plant pathogen, opportunistic animal pathogen | genomic/1 | DOI: 10.1094/PHYTO-08-16-0286R | WP_003481344.1 |
| *Xanthomonas vasicola* | soil, aquatic | plant pathogen, opportunistic animal pathogen | genomic/1 | DOI: 10.1111/j.13653059.2009.02124.x | WP_039446411.1 |

**Table S2** The most significantly enriched pathways among the genes in direct vicinity of avidin gene.

| \| **GO-term** \| **N_avid_** \| **N_total_** \| **Odds ratio** \| **P-value** \| **GO-function** \| \| --- \| --- \| --- \| --- \| --- \| --- \| \| GO:0047632 \| 1 \| 2 \| 562.17 \| 0.0027 \| agmatine deiminase activity \| \| GO:0005540 \| 1 \| 3 \| 374.78 \| 0.0036 \| hyaluronic acid binding \| \| GO:0000150 \| 3 \| 343 \| 9.95 \| 0.0038 \| recombinase activity \| \| GO:0003933 \| 1 \| 8 \| 140.54 \| 0.0080 \| GTP cyclohydrolase activity \| \| GO:0009446 \| 1 \| 8 \| 140.54 \| 0.0080 \| putrescine biosynthetic process \| \| GO:0000156 \| 2 \| 152 \| 14.88 \| 0.0086 \| phosphorelay response regulator activity \| \| GO:0004474 \| 1 \| 9 \| 124.92 \| 0.0089 \| malate synthase activity \| \| GO:0006097 \| 1 \| 9 \| 124.92 \| 0.0089 \| glyoxylate cycle \| \| GO:0004668 \| 1 \| 10 \| 112.43 \| 0.0098 \| protein-arginine deiminase activity \| \| GO:0006323 \| 1 \| 10 \| 112.43 \| 0.0098 \| DNA packaging \| \| GO:0019217 \| 1 \| 10 \| 112.43 \| 0.0098 \| regulation of fatty acid metabolic process \| \| GO:0004803 \| 3 \| 527 \| 6.47 \| 0.0123 \| transposase activity \| \| GO:0000062 \| 1 \| 14 \| 80.30 \| 0.0133 \| fatty-acyl-CoA binding \| \| GO:0000334 \| 1 \| 14 \| 80.30 \| 0.0133 \| 3-hydroxyanthranilate 3,4-dioxygenase activity \| \| GO:0006313 \| 3 \| 545 \| 6.26 \| 0.0134 \| transposition, DNA-mediated \| \| GO:0016779 \| 2 \| 220 \| 10.28 \| 0.0172 \| nucleotidyltransferase activity \| \| GO:0051607 \| 1 \| 20 \| 56.21 \| 0.0186 \| response to virus \| \| GO:0004565 \| 1 \| 27 \| 41.64 \| 0.0248 \| β-galactosidase activity \| \| GO:0030145 \| 1 \| 27 \| 41.64 \| 0.0248 \| manganese ion binding \| \| GO:0004497 \| 1 \| 31 \| 36.26 \| 0.0282 \| monooxygenase activity \| \| GO:0006858 \| 1 \| 31 \| 36.26 \| 0.0282 \| extracellular transport \| \| GO:0010309 \| 1 \| 31 \| 36.26 \| 0.0282 \| acireductone dioxygenase [iron(II)-requiring] activity \| \| GO:0006355 \| 10 \| 5323 \| 2.18 \| 0.0300 \| regulation of transcription, DNA-templated \| \| GO:0043571 \| 1 \| 33 \| 34.06 \| 0.0300 \| maintenance of CRISPR repeat elements \| \| GO:0019012 \| 1 \| 36 \| 31.23 \| 0.0326 \| virion \| \| GO:0006310 \| 3 \| 852 \| 3.99 \| 0.0420 \| DNA recombination \| \| GO:0005976 \| 1 \| 50 \| 22.48 \| 0.0446 \| polysaccharide metabolic process \| \| GO:0005506 \| 2 \| 417 \| 5.42 \| 0.0547 \| iron ion binding \| \| GO:0050518 \| 1 \| 62 \| 18.13 \| 0.0548 \| 2-C-methyl-D-erythritol 4-phosphate cytidylyltransferase activity \| \| GO:0004177 \| 1 \| 65 \| 17.29 \| 0.0574 \| aminopeptidase activity \| \| GO:0004499 \| 1 \| 73 \| 15.40 \| 0.0641 \| N,N-dimethylaniline monooxygenase activity \| \| GO:0016846 \| 1 \| 76 \| 14.79 \| 0.0666 \| carbon-sulfur lyase activity \| \| GO:0020037 \| 2 \| 552 \| 4.09 \| 0.0886 \| heme binding \| \| GO:0008299 \| 1 \| 105 \| 10.70 \| 0.0906 \| isoprenoid biosynthetic process \| \| GO:0009975 \| 1 \| 107 \| 10.50 \| 0.0922 \| cyclase activity \| \| GO:0045735 \| 1 \| 107 \| 10.50 \| 0.0922 \| N2-acetyl-L-aminoadipate semialdehyde dehydrogenase activity \| \| GO:0004519 \| 1 \| 109 \| 10.31 \| 0.0938 \| endonuclease activity \| \| GO:0009966 \| 1 \| 111 \| 10.12 \| 0.0954 \| regulation of signal transduction \| | |
| --- | --- | --- | --- | --- | --- | --- | --- | --- | --- | --- | --- | --- | --- | --- | --- | --- | --- | --- | --- | --- | --- | --- | --- | --- | --- | --- | --- | --- | --- | --- | --- | --- | --- | --- | --- | --- | --- | --- | --- | --- | --- | --- | --- | --- | --- | --- | --- | --- | --- | --- | --- | --- | --- | --- | --- | --- | --- | --- | --- | --- | --- | --- | --- | --- | --- | --- | --- | --- | --- | --- | --- | --- | --- | --- | --- | --- | --- | --- | --- | --- | --- | --- | --- | --- | --- | --- | --- | --- | --- | --- | --- | --- | --- | --- | --- | --- | --- | --- | --- | --- | --- | --- | --- | --- | --- | --- | --- | --- | --- | --- | --- | --- | --- | --- | --- | --- | --- | --- | --- | --- | --- | --- | --- | --- | --- | --- | --- | --- | --- | --- | --- | --- | --- | --- | --- | --- | --- | --- | --- | --- | --- | --- | --- | --- | --- | --- | --- | --- | --- | --- | --- | --- | --- | --- | --- | --- | --- | --- | --- | --- | --- | --- | --- | --- | --- | --- | --- | --- | --- | --- | --- | --- | --- | --- | --- | --- | --- | --- | --- | --- | --- | --- | --- | --- | --- | --- | --- | --- | --- | --- | --- | --- | --- | --- | --- | --- | --- | --- | --- | --- | --- | --- | --- | --- | --- | --- | --- | --- | --- | --- | --- | --- | --- | --- | --- | --- | --- | --- | --- | --- | --- | --- | --- | --- | --- | --- | --- | --- | --- | --- | --- | --- | --- | --- | --- |
|  |  |

**Table S3** Prediction of the structure-function of extended avidins. Domain prediction algorithms suggest the presence of both an avidin-like domain and an aspartyl protease-like domain in some of the bacterial putative avidin sequences. The predicted protein families predicted by Pfam (<https://pfam.xfam.org/>, Sonnhammer et al. 1997), Conserved Domain Database (CDD, <https://www.ncbi.nlm.nih.gov/Structure/cdd/wrpsb.cgi>; Marchler-Bauer & Bryant 2004) and InterPro (<https://www.ebi.ac.uk/interpro/search/sequence/>; Hunter et al. 2009) are shown with their associated E-values.

|  | avidin | | | protease | | |
| --- | --- | --- | --- | --- | --- | --- |
|  | Pfam | CDD | InterPro | Pfam | CDD | InterPro |
|  | Avidin family | Avidin superfamily | Avidin-like superfamily | Eukaryotic aspartyl protease | various | Aspartic peptidase domain superfamily |
| *Cytophagales bacterium 1* | 6.6e-17 | 1.8e-16 | 2.2e-24 | 8.3e-35 | pepsin_like: 6.4e-27 | 7.8e-46 |
| *Flexibacter roseolus* | 5.2e-11 | 1.1e-06 | 7.3e-18 | 1.6e-38 | Asp superfamily: 3.0e-32 | 1.1e-48 |
| *Nitrincola sp. AK43* | 8.5e-19 | 3.7e-16 | 2.6e-25 | 3.6e-33 | pepsin-like: 1.7e-24 | 6.0e-42 |
| *Oleiagrimonas soli* | 2.4e-14 | 3.0e-12 | 3.5e-21 | 2.7e-28 | pepsin-retropepsin-like: 3.8e-27 | 3.9e-37 |
| *Pseudomonas fluorescens* | 2.7e-07 | 7.0e-07 | 1.6e-16 | 1.0e-18 | pepsin-like: 8.9e-24 | 2.2e-36 |
| *Pseudomonas veronii* | 5.4e-06 | 2.4e-04 | 4.6e-16 | 6.4e-14 | pepsin-like: 2.9e-17 | 1.3e-32 |

**Table S4** Pairwise identities for the representative avidin sequences. Pairwise sequence identity (upper right part) and pairwise sequence similarity (lower left part) calculated using MatGAT 2.0 program (Matrix Global Alignment Tool). The calculation was performed using the default settings of the MatGAT 2.0 utilizing BLOSUM50 matrix. The resulting similarity/identity matrix has been gradient colored using white for low values and green color for high values.


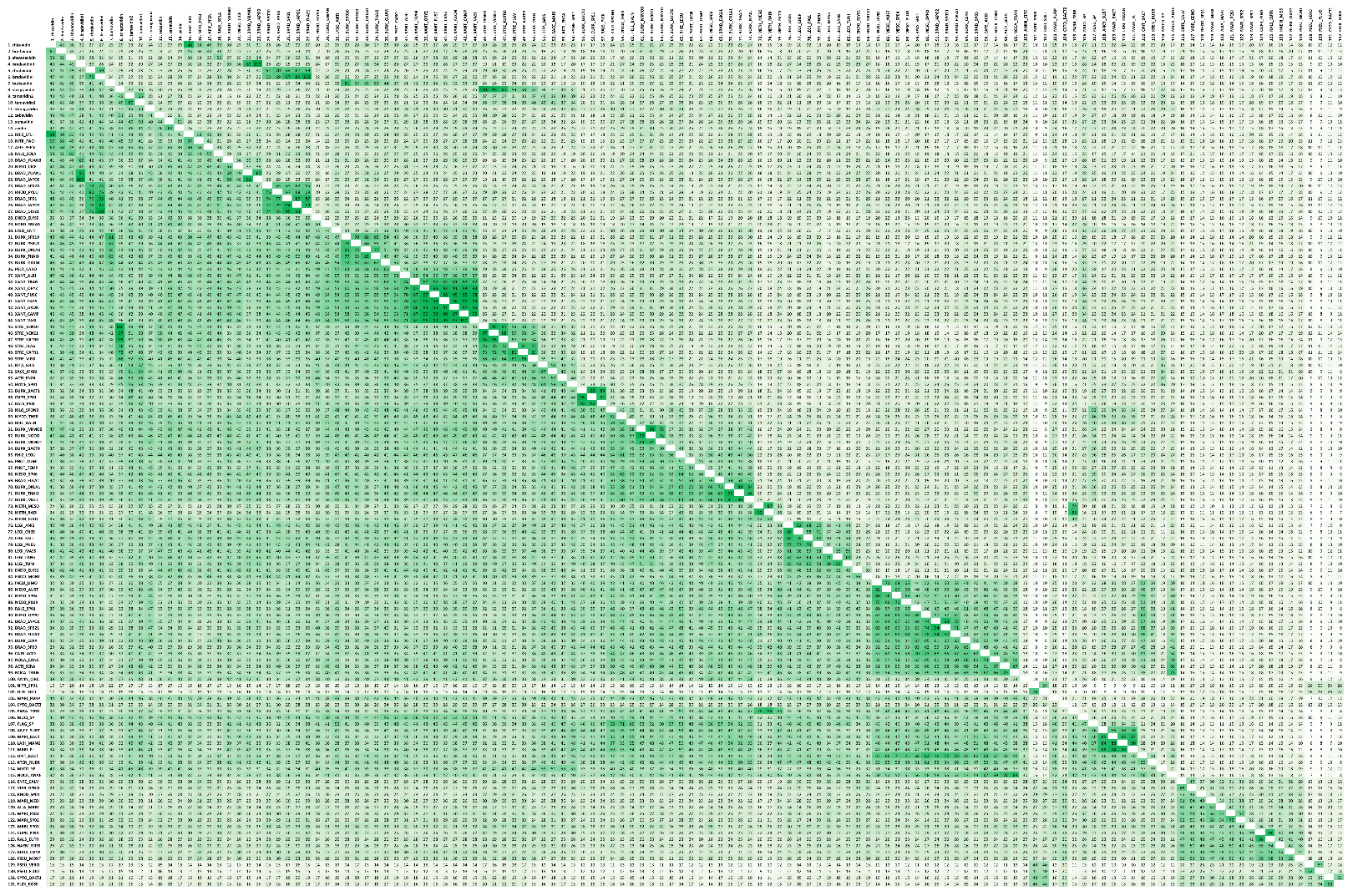


**Supplementary references**

Marchler-Bauer A, Bryant SH. CD-Search: protein domain annotations on the fly. Nucleic Acids Res. 2004;32(Web Server issue):W327-31. doi: 10.1093/nar/gkh454

Sonnhammer EL, Eddy SR, Durbin R. Pfam: a comprehensive database of protein domain families based on seed alignments. Proteins. 1997;28:405-20. doi: 10.1002/(sici)1097-0134(199707)28:3<405::aid-prot10>3.0.co;2-l

Hunter S, Apweiler R, Attwood TK, Bairoch A, Bateman A, Binns D, Bork P, Das U, Daugherty L, Duquenne L, Finn RD, Gough J, Haft D, Hulo N, Kahn D, Kelly E, Laugraud A, Letunic I, Lonsdale D, Lopez R, Madera M, Maslen J, McAnulla C, McDowall J, Mistry J, Mitchell A, Mulder N, Natale D, Orengo C, Quinn AF, Selengut JD, Sigrist CJ, Thimma M, Thomas PD, Valentin F, Wilson D, Wu CH, Yeats C. InterPro: the integrative protein signature database. Nucleic Acids Res. 2009;37(Database issue):D211-5. doi: 10.1093/nar/gkn785

Campanella JJ, Bitincka L, Smalley J. MatGAT: an application that generates similarity/identity matrices using protein or DNA sequences. BMC Bioinformatics 2003;4:29. doi: 10.1186/1471-2105-4-29
